# Facilitating a change model in age-friendly hospital certification: Strategies and effects

**DOI:** 10.1371/journal.pone.0213496

**Published:** 2019-04-03

**Authors:** Ying-Ling Kuo, I-Ju Chen

**Affiliations:** 1 Department of Nursing, Yonghe Cardinal Tien Hospital, Taipei, Taiwan; 2 Department of Nursing, National Yang-Ming University, Taipei, Taiwan; University of Malta Faculty of Health Sciences, MALTA

## Abstract

The ageing population is a powerful and transformative demographic force. The World Health Organization (WHO) has encouraged the development of an age-friendly hospital (AFH) network. However, no specific implementation strategies or best practices of AFH standards have been produced. This study sought to apply Kotter’s change model to the elements included in a successful AFH certification process and to evaluate the changes in employees’ knowledge of ageing and their attitudes toward the elderly. This was an observational study that utilized a pre- and posttest design, before and after an age-friendly hospital certification process was implemented. Participants were 163 hospital employees in Taiwan, who completed both pre- and postquestionnaires. The self-administered online questionnaire consisted of three sections: The Facts on Ageing Quiz, the Geriatric Attitudes Scale, and a demographic questionnaire. Following introduction of the intervention, the change process began, and later Kotter’s model was brought in as a descriptive framework. The results showed that Kotter’s eight-step framework is a good choice for thinking about how to change practice and make healthcare more age-friendly. Employee knowledge of ageing and their attitudes toward the elderly improved after this certification process. Appointing a chief executive officer, forming a steering committee, obtaining interdepartmental and interdisciplinary cooperation, and “soliciting support” for new policies from all employees, were identified as key factors influencing the success of age-friendly hospital (AFH) certification. This is the first study to apply Kotter’s eight-step framework of organizational change to an AFH certification process.

## Introduction

The ageing population is a powerful and transformative demographic force. In 2015, the number of elderly individuals (over 65 years) worldwide was 901 million, comprising 12.3% of the total population [1]. By March 2018, the proportion of elderly population in Taiwan reached 14.05%, officially entering the “Aged Society.” The World Health Organization (WHO) has advocated the importance of developing age-friendly environments by introducing the principles of proactive age-friendly healthcare [2]. In particular, the WHO World Report on Ageing and Health provides a framework to guide countries in taking concrete actions toward improving the lives of the elderly worldwide [3]. The International Network of Health Promoting Hospitals and Health Services supports the development of “Age-friendly Health Care” [4] to address the specific needs and capabilities of different patient groups for health promotion in hospitals and health services. Specific standards and self-assessment tools for particular target groups have been developed—for “Health Promotion for Children & Adolescents in and by Hospitals,” for “Age-friendly Health Care” [5], and for “Migrant-friendly and Culturally Competent Healthcare.” The WHO has also encouraged the development of an Age-Friendly Hospital (AFH) care network [6,7], defined as a system that can accommodate the elderly by providing barrier-free mobility and enhancing active ageing [6,7]. Providing an appropriate, pleasant, supportive, respectful, and accessible AFH for elderly patients is a critical component of the age-friendly environment [2]. Therefore, the mission of an AFH should be to provide a comprehensive and appropriate care environment for elderly [8,9]. However, no specific implementation policies or best practices of AFH standards have been produced.

Taiwan is ageing faster than European and North American countries [1], and there is an urgent need to build an AFH network. Thus, the Health Promotion Administration, Ministry of Health and Welfare, Taiwan, proposed the Taiwan Framework for AFH (TFAFH) and certification standards, which are based on the WHO’s age-friendly care principles [6,7]. TFAFH is the first officially approved AFH certificate program [9,10]. TFAFH comprises the vision, values, missions, and strategies across four dimensions of an AFH: management policy, communication and services, physical environment, and care processes [4,9]. The certification standards comprise 60 measurable items across 4 primary standards and 11 substandards [5,9,10] (Table 1). Standard 1 “Management policy” includes developing an age-friendly policy, organizational support, and continuous monitoring and improvement. Standard 2 “Communication and services” covers staff, information provision, respect for senior decision-making, administrative support, and volunteer participation. Standard 3 “Care processes” includes patient assessment, intervention, and management (guidelines for geriatric assessment and interventions on high-risk seniors are available), community partnership and continuity of care. Standard 4 “Physical environment” includes the hardware environment (universal design), traffic and action, signs, and identification. A certification committee reviews submitted AFH applications and conducts on-site hospital visits following the review. The AFH certificate must be renewed every four years.

**Table 1:** Associations between Kotter’s change model and the process of age-friendly hospital certification.

Kotter’s 8-step change model		Process and associated standards of age-friendly hospital certification (AFH certification)	
Steps	Suggested strategies/actions	Strategy applied to achieve AFH certification standards	AFH certification standards
Step 1. Create a sense of urgency	• Examine market and competitive realities. • Provide evidence from outside the organization that change is necessary. • Identify and discuss crisis, potential crisis, or major opportunities.	• Conduct SWOT analysis - The case study hospital (Health Promotion Committee) collected information on SWOT and analyzed it prior to making the decision to apply for AFH certification.	Standard 1. Management policy • developing an age-friendly policy
		• The Health Promotion Committee confirms, declares, and formulates policies through internal or supervisory communication.	Standard 1. Management policy • organizational support
Step 2. Build the guiding team	• Assemble a group with enough power to lead the change effort. • Attract key leaders of change by showing enthusiasm and commitment. • Encourage the group to work together as a team.	• The steer committee was constituted. The 20 members invited to join were superintendents, directors of medical and administrative departments, nursing supervisors, medical professional representatives, clerks, aged patients, family caregivers, and volunteers.	Standard 1. Management policy • organizational support
		• A nursing supervisor was appointed to be the CEO. • The PDCA method was employed to develop, implement, and evaluate the process of AFH certification.	Standard 2. Communication and services • communication Standard 1. Management policy • continuous monitoring and improvement
Step 3. Create a vision	• Create a vision to help direct the change effort. • Develop strategies for achieving that vision.	• Collective decision making • Build consensus - All employees were broadly engaged in the entire conversation, which consisted of “collective decision making (across the hospital), consensus building, and strategy formulation for the vision.”	Standard 2. Communication and services • communication • services (internal customers: employees; external customers: patients, family, and community)
		• Formulate a strategy for the vision The teams were encouraged to develop a variety of communication channels to convey the authentication steps and strategies. • Pretest questionnaire Pretest questionnaires were administered at this stage.	Standard 1. Management policy • continuous monitoring and improvement
Step 4. Communicate the vision	• Build alignment and engagement through stories. • Use every vehicle possible to communicate the new vision and strategies. • Keep communication simple and heartfelt. • Teach new behaviors by the example of the guiding coalition.	• The common vision—“love God, love people, and respect life—age-friendly”—was shared with staff. • The CEO led internal marketing efforts. • Internal marketing communication - The internal marketing communication approach focused on short- to medium-range targets, including possible communication channels, large and small conferences in hospitals, websites, publications within the hospital, regular e-mail bulletins, etc. - Multiple communication channels ensured a common vision for the hospital staff. - Authorized employee participation and a distress strategy indicated positive attitudes toward employees.	Standard 2. Communication and services • communication services (internal customers: employees; external customers: patients, family, and community)
		• Education (workshop, continued education, experiential ageing-simulation learning, etc.) - To meet the healthcare needs of elderly, workshops, continued education, and ageing-experience activities were conducted to ensure hospital-wide participation in the comprehensive review of operational procedures, the overall environment, and equipment.	Standard 2. Communication and services • communication • services
		- The training includes 2 hours of age-friendly education (continuing education) and 3 hours of experiential ageing-simulation activity (Including the necessary courses for on-the-job and new employee training).	Standard 4. Physical environment • hardware environment: “universal design” • traffic and action • signs and identification
		- The method and content of communications between hospital workers, elderly, and the service processes needed to be improved to establish friendly, respectful, and accessible services for elderly in accordance with communication and service standards.	Standard 2. Communication and services • communication • services
Step 5. Empower action	• Remove obstacles to change. • Change systems or structures that work against the vision.	• Multidisciplinary team - This step required the cooperation of the professional teams; therefore, interdisciplinary cooperative strategies were implemented. • The CEO convened a meeting every 2 weeks to discuss policies and measures in such areas as general affairs, medical affairs, and health communications to unite outpatient nursing staff and administrative personnel. - The CEO convened a meeting with nursing supervisors, nutritionists, physical therapists, and mental health physicians, who jointly formulated the following measure: the reevaluation of older inpatient assessment forms in areas including physical function, mental status, nutritional screening, and fall risks, and the integration of all forms into one high-risk screening form. • Patient support groups - Service desks and service windows were established for elderly so they could receive priority appointment services. - Service processes were emphasized, such as reducing wait times and keeping the sixth appointment number for an elderly patient. - Elderly were provided with information regarding convenient transportation, reserved seating, extra space for entering and exiting vehicles, calling cabs for patients, and shuttle buses. - A website was created, giving patients (family caregivers) and staff access to “health information on health ageing, risk factors, and diseases.” - Improvements in older adult communication were achieved through the use of larger fonts and clearer diagrams in health education tools and pamphlets and the use of other age-appropriate methods, such as styluses and electronic communicators. - Emphasis was placed on respect for elderly in all care processes by proactively informing them of their diagnoses, examination results, treatments, and conditions. They were physically able to, as well as obtaining informed consent from self-aware elderly. - Community resources and patient support groups were united to meet the continual care standard.	Standard 3. Care processes • patient assessment • intervention and management (guidelines on multidisciplinary geriatric assessment and interventions on high-risk seniors are available) Standard 4. Physical environment • hardware environment: “universal design” • traffic and action • signs and identification Standard 2. Communication and services • communication • services Standard 3. Care processes • community partnership and continuity of care (a list of health and social care providers working in partnership with the hospital is available)
Step 6. Create short-term wins	• Plan for and achieve visible performance improvements. • Recognize and reward those involved in bringing the improvements to life.	• Mobilization - A comprehensive review and authorized employee participation (mobilization) of the hospital environment was conducted. - When the goals have been met, the employees will be motivated to fine tune and expand the change. •Strategies are developed for short-term wins. - By acknowledging and rewarding employees who are closely involved in the change process, it will be clear across the board that the company is changing course.	Standard 4. Physical environment • hardware environment: “universal design” • traffic and action • signs and identification
Step 7. Build on the changes	• Plan for and create visible performance improvements. • Recognize and reward personnel involved in the improvements. • Reinforce the behaviors shown that led to the improvements.	• Repair decoration The hospital environment was improved based on two general principles: universal design and barrier-free issues related to ageing. - For example, considering the safety of elderly entering and exiting hospital rooms, the colors brown and yellow, which are not easily distinguishable, were eliminated from all hospital rooms and facilities. - Larger fonts and contrasting colors were used on all signage within the hospital; reception chairs were replaced with those with armrests; the slope was adjusted on all ramps, on which nonslip strips were also installed; and warning signs were posted.	Standard 4. Physical environment • hardware environment: “universal design” • -traffic and action • -signs and identification
		• Posttest questionnaire - The posttest questionnaire was administered at this stage.	Standard 1. Management policy • continuous monitoring and improvement
Step 8. Institute the change	• Articulate the connections between the new behaviors and corporate success.	• Certification success - The hospital received AFH certification for the first time on September 3, 2012, and was recertified to 2021 in 2015. • Intercollegiate exchange visit - Intercollegiate exchange visit took place on March 29, 2016.	Standard 1. Management policy • continuous monitoring and improvement

Note: AFH = age-friendly hospital; SWOT = strength, weakness, opportunity, threat; PDCA = plan-do-check-act.

Achieving AFH certification is a process of change, and managing change is an important and difficult task for health leaders. Organizations, individuals, and communities get caught in “comfort zones,” and a substantial obstacle to change in organizations is “getting people to change.” Kotter proposed the change model and guidelines to manage how to successfully complete a change [11]. Kotter’s change model emphasizes the buy-in of employees as the focus for success; it is important to provide clear steps to guide the process that are easy to understand [11]. John Kotter’s eight steps of change include Step 1, “create a sense of urgency.” The transformation suggestions include examining the market and competitive realities; identifying and discussing crises, potential crises, or major opportunities; and providing evidence from outside the organization. The second step, “build a guiding team,” involves assembling a group with enough power to lead the change effort, attracting key leaders of change by showing enthusiasm and commitment, and encouraging the group to work together as a team. Step 3, “creating a vision,” aims to help direct the change effort and development strategies for achieving that vision. The fourth step, “communicate the vision,” includes building alignment and engaging through stories, using every vehicle possible to communicate the new vision and strategy, maintaining simple and genuine communication, and teaching new behaviors by using the example of the guiding coalition. The fifth step, “enable action,” involves removing obstacles to the change and changing the systems or structures that work against the vision. In step 6, the strategy is to “create short-term successes,” plan for and achieve visible performance improvements, and recognize and reward those involved in bringing the improvements to life. Step 7 is “build on the change,” which is defined as not giving up, planning for and creating visible performance improvements, recognizing and rewarding personnel involved in the improvements, and reinforcing the behaviors that led to the improvements. The final step is to “institute the change,” in which the connections between new behaviors and corporate success are articulated and made permanent.

Employee knowledge and attitudes toward the policy are the key components of successful change [12,13]. Hsiao et al. (2011) reported that efficient procedures as well as well-prepared hospital staff play an important role in the implementation policies [14]. Thus, the knowledge and attitudes of employees toward ageing are important for successful AFHs. Currently, the promotion of age-friendly environments and healthcare facilities remains in the initial stages [15,16]. Thus, bridging the gap between vision and practice is urgently needed to satisfy the needs of ageing populations [17]. The process of change affects the institution itself and the individuals in it. At the same time, through certification, the staff’s changes in knowledge and attitudes have an impact on the change process in the organization and the individual staff. The current study sought to describe the certification process of an AFH using John Kotter’s change model and to evaluate the changes in employees’ knowledge of ageing and their attitudes toward elderly.

## Methods

### Research design, setting, and participants

This was an observational study with a pre- and posttest design applied to measure the changes following an AFH certification process. A teaching hospital founded in 1981 in northern Taiwan and certified as a member of the WHO Health Promoting Hospital (HPH) Network in 2006 was the subject of this study. The hospital had 342 beds and 615 employees; 54.3% (n = 334) were nurses, 25.25% (n = 157) were physicians and other medical personnel, and the remaining 25.2% (n = 124) were administrative staff. After eliminating 31 ineligible employees who were training abroad, on parental leave, or had requested a leave of absence, 584 employees were recruited for this study. A self-administered online questionnaire was used to collect the data.

### Procedures

This study was approved by the Ethics Committee of Cardinal Tien Hospital (CTH-101-3-5-047). The anonymity of participants was preserved, and no financial burden was imposed on them. The researchers participated in, observed, and recorded the certification process, and John Kotter’s change model was applied to describe the certification process. The pretest questionnaire was administered from July 6 to July 30, 2012, prior to the AFH certification visit (September 3, 2012). The posttest questionnaire was administered from December 17, 2012, to January 12, 2013.

### Measures

The questionnaire consisted of five sections, including demographics, experiences with caring for the aged, the Facts on Ageing Quiz (FAQ1), the Geriatric Attitudes Scale (GAS), and the open question (comments).

#### Demographics, experience with caring for the aged

The individual-related variables included age, marital status, family composition, experience with caring for elderly family members, duration of care for the aged, feelings when interacting with the elderly, and occupational categories. The items for “feelings when interacting with the elderly” were scored across three dimensions: 1 = nothing specific, 2 = fair, and 3 = good.

#### First Facts on Ageing Quiz (FAQ1)

Palmore designed the FAQ1 to assess knowledge of physical, psychological, and social factors related to ageing and measure misconceptions regarding elderly [18]. The Chinese version of the FAQ1 was translated with the original author’s permission [19,20]. Two bilingual translators translated the original scale into the target language independently and then met to discuss their translations. One bilingual translator then performed the back-translation, and an English-speaking person with a cross-cultural background compared the original and back-translated English versions for conceptual equivalence. In this procedure, if the two scales were not equivalent, the differences were explained to the bilingual translators, after which the same group of translators and experts repeated the procedure until the forward- and back-translated scales were equivalent [19,20]. The 25 items of this scale encompass 7 items regarding physical concerns, 7 items regarding psychological concerns, and 11 items regarding the social dimensions of ageing and common misconceptions about ageing [19,21,22]. The scale comprises true–false questions and has been previously used in studies with college students, in partial groups of hospital workers, and in geriatrics education [23,24]. Palmore’s quizzes are used most often to measure changes in knowledge resulting from some educational intervention in college students [18,19,21,22]. There have been other studies using this tool for before and after comparisons [20,25].

Correct answers were assigned 4 points, and incorrect answers were assigned 0 points, with the total score ranging from 0 to 100 points and a higher score indicating greater knowledge of ageing [23–27]. Previous studies have found the Cronbach’s α coefficient to be between 0.68 and 0.82 [18,19,21,22].

#### Geriatric Attitudes Scale (GAS)

The GAS has been commonly used in previous studies [28–32]. The Chinese version of the GAS was translated with the original author’s permission [20,29]. The GAS has been used internationally, often with modifications [32]. For the GAS, a Chinese translation version was provided by Dr. Ming Lee [29], one of the designers. Cronbach’s α in the original work was 0.76, with good construct validity [29].

There are 14 statements in the GAS, which are scored across 5 dimensions: 1 = strongly disagree, and 5 = strongly agree. The GAS makes use of five positive and nine negative statements about elderly [32]. Scores were added and divided by 14 to obtain an average score, with a higher score indicating a more positive attitude toward elderly and an average score of 3 or higher considered a positive attitude [28,29,30]. The Cronbach’s α coefficient for the GAS has been found to be between 0.69 and 0.76 [28,29,30,31,32].

#### Open question (comments)

In the last section of the survey, an open question was presented to staff to determine their feelings about certification and the change to an age-friendly hospital.

### Data analysis

Statistical analyses were performed using SPSS Version 20. Percentage, mean, and standard deviation were used to describe the demographic characteristics, experience with elderly, work status, knowledge of ageing, and attitude toward elderly. A paired *t*-test was used to assess variations in the hospital employees in terms of knowledge of ageing and attitudes toward elderly before and after the implementation of AFH certification policies. All analyses of significance employed two-tailed *t*-tests with *α* = 0.05.

## Results

### Applying the change model to describe the AFH certification process and strategies

Between July 1, 2012, and December 1, the certification intervention was introduced and the change process occurred; later, Kotter’s model was brought in as a descriptive framework to study the process and outcomes. Kotter’s eight-step process and transformation suggestions were used to compare and describe the AFH certification process and strategies for change outlined in the following sections (Table 1).

#### Step 1. Create urgency

The plan-do-check-act (PDCA) approach is used extensively for quality management in various fields; it is a process of finding and solving problems, and is applicable to making continuous improvements in medical quality management [33,34]. The case study hospital (Health Promotion Committee) conducted a strengths, weaknesses, opportunities, and threats (SWOT) analysis prior to the decision to seek AFH certification. The PDCA method was employed to ensure that all planned certification strategies and preparations conformed to the four standards outlined in “Taiwan’s Framework for Age-Friendly Hospitals.” The case study hospital (Health Promotion Committee) confirms, declares, and formulates policies through internal or supervisory communication. They examined market and competitive realities; identified and discussed crises, potential crises, and major opportunities; and provided evidence from outside the organization that change was necessary.

#### Step 2. Build the guiding team

To ensure that organized support was available to meet the management policy standards, an age-friendly steering committee was formed, which was responsible for four tasks: developing policies (making specific regulations), soliciting support for policies from all employees, formulating execution strategies that promote hospital-wide participation in these policies, and tracking and monitoring the execution strategies. The chief of staff served as the chairperson of this committee and appointed the nursing supervisor to act as the chief executive officer (CEO) of the certification process. The CEO was responsible for formulating interdepartmental and interdisciplinary cooperation policies, executing the strategies, and negotiating, coordinating, advocating, and monitoring policies. The age-friendly steering committee members included the director of the hospital, directors of departments (Medical Department, Department of Nursing, Community Medicine Department, Family Medicine Department, General Affairs Office, Public Works Office, Social Service Office, etc.), the Nursing Department supervisor, professional leaders (nutrition, occupational therapy, physical therapy, speech-language pathology, etc.), grassroots employees (clinical nurses, community nurses, outpatient nurses, registered personnel), an elderly patient representative, a family caregiver’s representative, and a hospital volunteer.

#### Step 3. Create a vision

All employees were broadly engaged in the entire conversation, which consisted of “collective decision making (across the hospital), consensus building, and strategy formulation for the vision.” The pretest questionnaires were administered at this stage. The teams were encouraged to develop a variety of communication channels to convey the authentication steps and strategies.

#### Step 4. Communicate the vision

The common vision, “Love God, love people, and respect life—age-friendly,” was shared with staff. The CEO led the internal marketing efforts. The internal marketing communication approach focused on short- to medium-range targets, including possible communication channels, large and small conferences in hospitals, websites, publications within the hospital, regular e-mail bulletin progress, and so forth. Multiple communication channels ensured a common vision for the hospital staff. Authorized employee participation and a distress strategy demonstrated positive attitudes toward employees.

To meet the healthcare needs of the elderly, six workshops, continued education (one time, two hours), and an experiential ageing-simulation activity (one time, three hours) were conducted to ensure hospital-wide participation in the comprehensive review of operational procedures, the overall environment, and equipment.

The workshops included two hours of age-friendly classes and three hours of elderly simulation programs. The training education included two hours of age-friendly classes; the content focused on understanding changes in the body, mind, and spirit of the elderly.

The experiential ageing-simulation activity lasted three hours. Employees wore earplugs, glasses with yellow lenses, kneepads, ankle guards, gloves, and aggravated vests to experience the limitations the elderly experience in terms of hearing, vision, joint stiffness (knees, wrists, fingers), mobility, and being stooped. The method and content of communications between hospital workers and elderly patients regarding the service processes needed to be improved to establish friendly, respectful, and accessible services for elderly in accordance with communication and service standards.

#### Step 5. Empowering action

To realize the vision, interdepartmental collaboration (multidisciplinary teams, vertical integration, horizontal integration) between administrative and medical departments was implemented. The CEO convened a meeting every two weeks to jointly discuss policies and measures in such areas as general affairs, medical affairs, and health communications for uniting outpatient nursing staff and administrative personnel. Improvements in older adult communication were achieved by using larger fonts and clearer diagrams in health education tools and pamphlets and the use of other age-appropriate methods, such as styluses and electronic communicators. A website was created for patients, family caregivers, and staff to have access to “health information on health ageing, risk factors, and diseases.” Information was provided regarding convenient transportation, reserved seating, extra space for entering and exiting vehicles, calling cabs for patients, and shuttle buses. Service desks and service windows were established for seniors so they could receive priority appointment services. Service processes were emphasized, such as reducing waiting times, keeping the sixth appointment number for an elderly patient, and eliminating outpatient copayments for patients aged 100 years or above, to reduce the burden of medical expenses.

Older patient assessment forms needed to be integrated, continuous care needed to be jointly provided, and professional care process uniformity needed to be achieved to provide the effective and safe care required to meet care process standards. This step required the cooperation of the professional teams; therefore, interdisciplinary cooperative strategies were implemented. The CEO convened a meeting with nursing supervisors, nutritionists, physical therapists, and mental health physicians, who jointly formulated the following measure: the reevaluation of older inpatient assessment forms for physical function, mental status, nutritional screening, and fall risks, and the integration of all forms into one high-risk screening form for seniors. Community resources and patient support groups were united to meet the continual care standards. Emphasis was placed on respect for the elderly in all care processes by proactively informing them of their diagnoses, examination results, treatments, and conditions, as well as obtaining informed consent from self-aware elderly, if possible.

#### Step 6. Create short-term wins

A comprehensive review of the hospital environment was conducted with authorized employee participation. “Nothing motivates more than success. Give your company a taste of victory early in the change process. Within a short time frame (this could be a month or a year, depending on the type of change), you’ll want to have some ‘quick wins’ that your staff can see.” [11]. Most people will not go the distance unless they see compelling evidence that the journey is producing expected results in 12 to 24 months. When goals are met, employees will be motivated to fine tune and expand the change. By acknowledging and rewarding employees who are closely involved in the change process, it will be clear across the board that the company is changing course.

#### Step 7. Build on the change

The hospital environment was improved based on two general principles: universal design and barrier-free issues related to ageing. For example, considering the safety of elderly entering and exiting hospital rooms, the colors brown and yellow, which are not easily distinguishable, were eliminated from all hospital rooms and facilities; larger fonts and contrasting colors were used on all signage within the hospital; reception chairs were replaced with those with armrests; the slope was adjusted on all ramps, on which nonslip strips were also installed; and warning signs were posted. The posttest questionnaire was administered following this step.

#### Step 8. Institute the change

The hospital received AFH certification for the first time on September 3, 2012, and was recertified to 2021 in 2015. An intercollegiate exchange visit was conducted on March 29, 2016.

### Demographic characteristics and caring experiences of participants

The response rate for participating in the online surveys was 57.5% (n = 336) for the pretest and 44.2% (n = 258) for the posttest. A total of 163 participants completed both the pretest and posttest surveys: 27.9% were hospital employees, and 52.2% were nurses. A *t*-test and chi-squared goodness-of-fit test were employed to compare descriptive statistics of the sample and the case hospital’s population to test for selection bias. The resulting *p* values for gender, age, and work category were .28, .93, and .40, respectively, indicating that the sample was representative of the case hospital’s population.

Descriptive characteristics and care experience for the participating staff are presented in Table 2. The average age of participants was 39.25 years, and most participants were married and living with their nuclear families. The highest percentage of elder care experience was taking care of their own elderly family members (73.6%) (e.g., caring for a parent or grandparent). The most common duration of this care was less than one week. More than 50.3% of the participants rated their feelings when interacting with the elderly as “fair.”

**Table 2:** Demographic characteristics and care experiences of participants (n = 163).

Variables	*n*	%
Age		
<30 years	47	28.8
31–40 years	58	35.6
41–50 years	41	25.2
≥51 years	17	10.4
Marital status		
Married	93	57.1
Un-married	70	42.9
Family composition		
Three-generation extended family or more	34	20.9
Nuclear family	116	71.1
Single-parent families	13	8.0
Experience of caring for own elderly families (personal—e.g., caring for a parent or grandparent)		
Yes	120	73.6
No	43	26.4
Duration of care of the aged		
<1 week	106	65.0
≥1 week < 6 months	10	6.1
≥6 months	47	28.9
Feelings when interacting with the elderly		
Nothing specific	43	26.4
Fair	82	50.3
Good	38	23.3
Occupational categories		
Medical personnel	18	11.0
Nurse	85	52.2
Administrative staff	60	36.8

### Effect of AFH certification process on employees’ ageing knowledge

The ageing knowledge scores before and after the AFH certification process were 46.90 (SD = 8.99, range 28–76) and 54.55 (SD = 8.85, range 28–76), respectively. After the certification process, the accuracy of employees’ ageing knowledge increased significantly (*t* = 10.73, *p*<0.001), as shown in Table 3. Among the three dimensions of ageing, the highest scores were obtained for the category of physical knowledge (pretest accuracy was 68%; posttest accuracy was 80%). Scores on psychological knowledge (pretest and posttest scores were 43% and 52%, respectively) and social knowledge (pretest and posttest scores were 31% and 32%, respectively) were much lower. The results also revealed that although the overall knowledge of ageing after AFH certification significantly improved, the accuracy rate remained far below the standard of 60% [23–27]. Further analysis indicated that this improvement was concentrated in the physical (*t* = 10.77, *p*<0.001) and social (*t* = 8.67, *p*<0.001) knowledge areas.

**Table 3:** Employee knowledge of ageing and attitudes toward elderly before and after AFH certification (n = 163).

Variables	Pretest *M*±*SD (correct %)*	Posttest *M*±*SD (correct %)*	*t*
Facts on Ageing Quiz 1			
Overall	46.90±8.99 (46.90%)	54.55±8.85 (54.55%)	10.73***
Physical	19.12±3.75 (68%)	22.40±2.97 (80%)	10.77***
Psychological	8.83±3.70 (43%)	9.20±3.75 (52%)	1.32
Social	18.94±4.89 (31%)	22.92±4.93 (32%)	8.67***
Geriatric Attitude Scale	2.88±0.29	3.00±0.22	5.05***

Note: *M* = mean; *SD* = standard deviation;

*p<0.05,

**p<0.01,

***p<0.001.

### Effect of AFH certification strategies on employee attitudes toward elderly

Scores for employee attitudes toward elderly increased from 2.88 (SD = 0.29) before certification to 3.0 (SD = 0.22) after certification. These results were significant (*t* = 5.05, *p*<0.001), indicating that the AFH certification strategies had a significant positive effect on employee attitudes toward elderly.

### Results for the open question

The open question gave participants the opportunity to freely write out their responses. Their answers were classified in terms of how they related to “soliciting support” for new policies from all employees.

The results indicated that the participants considered the important factors to be soliciting support for new policies from all employees (n = 88), obtaining interdepartmental and interdisciplinary cooperation (n = 68), having a nurse manager as the CEO (n = 62), and forming a steering committee (n = 34).

## Discussion

The effect of the change intervention was actually demonstrated within six months of implementation of the intervention. The Guiding Coalition becomes a critical force in identifying significant improvements that can happen between 6 and 18 months [35]. Most people will not stay on board for the long term unless they see compelling evidence in 12 to 24 months that the journey is producing the expected results. Without short-term wins, too many people give up or join the ranks of those who resist change. For example, the new program was adopted about six months into the effort because it met multiple criteria [11]. The change usually takes 6 to 24 months [11,35], and our study was effective within 6 months of the intervention.

This is the first study to apply Kotter’s eight-step framework of organizational change in an AFH certification process. Kotter’s eight-step transformation framework and suggestions described the AFH certification process well and appropriately fit the certification strategies. Thus, our results indicate that this framework could serve as a reference for hospital leaders to institute organizational change, especially for age-friendly policies.

The hospital developed strategies to obtain official AFH certification, including appointing a nurse manager as the CEO, forming a steering committee, and obtaining interdepartmental and interdisciplinary cooperation. Studies have indicated that appointing one person to be solely in charge of monitoring the progression of policy objectives is a factor in the successful establishment of HPHs, and nurse managers are a preferred choice for such positions [13,14].

The formation of a steering committee was another key factor in the hospital’s certification as an AFH. Previous studies have recognized that hospitals must provide formal organizational support, necessary authority, and autonomy to realize organizational objectives and advanced management [14,36]. Hsiao *et al*. [14] also noted that one common factor for the successful establishment of an HPH is the formation of a supervisory committee.

We suggest that hospitals implementing changes should adopt interdepartmental and interdisciplinary cooperative strategies to effectively integrate services and raise the quality of healthcare services. In this study, hospital employees’ attitudes toward the elderly improved significantly as a result of the intervention [29].

However, we also found that knowledge of the psychological and social dimensions of ageing was weaker, which partially agreed with the results of other studies [19,20]. We speculate that questions about the elderly are often answered incorrectly because courses on ageing focus on the physical aspects of ageing. Finally, we suggest that in-service training should include courses in the social and psychological needs of the elderly. This change would improve employee communication with and attitudes toward elderly patients and create an age-friendly atmosphere.

## Conclusion

This study applied John Kotter’s change model to describe the AFH certification process and strategies. Kotter identified leadership (appointing a chief executive officer), forming a steering committee, obtaining interdepartmental and interdisciplinary cooperation, and “soliciting support” for new policies from all employees as key factors influencing the success of an organizational change plan [35]. The staff’s current level of understanding and attitudes and the impact of training experiences are important factors to consider when planning curricular changes to accomplish these objectives [25]. Because AFH certification was not completed the previous year, we interviewed our leaders to determine that these were the important factors. Further, results from the study indicated that forming a promotion committee, employing a nurse manager as the CEO, enacting interdepartmental and interdisciplinary cooperation, and soliciting support from all employees for new policies and decision-making strategies were key factors influencing the success of age-friendly care. Employee knowledge of ageing and their attitudes toward elderly improved after this certification process.

It may be surprising that after only six months of intervention, the organization was certified as an AFH. This could be explained by the fact that the organization was already an HPH. Yet, the posttest collection time was carried out three months after the certification. This may be the reason why the change in knowledge and attitudes before and after the intervention is relatively small.

Successful transformation in an organization may not be the same as achieving “certification” from the government as addressed in the discussion. To improve the health care system, we have changed our perspective on acute adults in order to provide appropriate and high-quality services for the elderly. However, whether it can be successful depends on making changes in the workplace culture. In addition to recertification after four years, it is necessary to look at other indicators (such as the satisfaction of senior citizens and their families) in the future. Since this research focused on hospital staff, there was no measurement from the perspective of the family caregiver; this is a limitation of this study. It is recommended that future studies include the measurement of family caregivers.

This study covered a timely and interesting topic—namely, the transformation of health care systems to better offer appropriate and high-quality services to older adults. There are several position papers on this topic but relatively little empirical research; scientific contribution is therefore highly valuable.

Kotter’s eight-step framework proved to be a successful tool for facilitating organizational change. The described processes and strategies serve can provide a reference for future hospitals or other HPH networks applying for AFH certification.
